# Iron deficiency anemia is not a rare problem among women of reproductive ages in Ethiopia: a community based cross sectional study

**DOI:** 10.1186/1471-2326-9-7

**Published:** 2009-09-07

**Authors:** Jemal A Haidar, Rebecca S Pobocik

**Affiliations:** 1 School of Public Health, Addis Ababa University, P.O. Box: 27285/1000, Addis Ababa, Ethiopia; 2 Ethiopian Health and Nutrition Research Institute, P.O. Box 5654, Addis Ababa, Ethiopia; 3 School of Family and Consumer Sciences, Bowling Green State University and Northwest Ohio Consortium for Public Health, 302 Johnston Hall, Bowling Green, OH, 43403-0059, USA

## Abstract

**Background:**

In Ethiopia, the existence of iron deficiency anemia is controversial despite the fact that Ethiopia is one of the least developed in Africa with a high burden of nutrient deficiencies.

**Methods:**

The first large nutrition study of a representative sample of women in Ethiopia was conducted from June to July 2005 and a systematically selected sub-sample of 970 of these subjects, 15 to 49 years old, were used in this analysis of nutritional anemia. Hemoglobin was measured from capillary blood using a portable HemoCue photometer. For serum ferritin, venous blood from antecubital veins was measured by an automated Elecsys 1020 using commercial kits. Diets were assessed via simplified food frequency questionnaire. The association of anemia to demographic and health variables was tested by chi-square and a stepwise backward logistic regression model was applied to test the significant associations observed in chi square tests.

**Results:**

Mean hemoglobin ± SD was 11.5 ± 2.1 g/dL with a 29.4% prevalence of anemia. Mean serum ferritin was 58 ± 41.1 ug/L with a 32.1% prevalence of iron deficiency. The overall prevalence rate of iron deficiency anemia was 18.0%. Prevalence of anemia, iron deficiency, and iron deficiency anemia was highest among those 31-49 years old (p < 0.05). Intake of vegetables less than once a day and meat less than once a week was common and was associated with increased anemia (p = 0.001). Although the prevalence of anemia was slightly higher among women with parasitic infestation the difference was not significant (p = 0.9). Nonetheless, anemia was significantly higher in women with history of illness and the association was retained even when the variable was adjusted for its confounding effect in the logistic regression models (AOR = 0.3; 95%CI = 0.17 to 0.5) signifying that the most probable causes of anemia is nutrition related and to some extent chronic illnesses.

**Conclusion:**

Moderate nutritional anemia in the form of iron deficiency anemia is a problem in Ethiopia and therefore, the need for improved supplementation to vulnerable groups is warranted to achieve the United Nation's Millennium Development Goals. Chronic illnesses are another important cause of anemia.

## Background

Anemia refers to a condition in which the hemoglobin content of the blood is lower than normal as a result of a deficiency of one or more essential nutrients [1], heavy blood loss, parasitic infections such as hookworm infestations, acute and chronic [2] infections, and congenital hemolytic diseases [2-5]. At least half of anemia worldwide is due to iron deficiency [6]. Iron deficiency is due primarily to a lack of bio-available dietary iron [7,8] or increased requirements such as during childhood and pregnancy [2]. Anemia increases risk for maternal and child mortality and has negative consequences on the cognitive and physical development of children, and on work productivity in adults [2]. Clinical signs of anemia include breathlessness, dizziness, and perceived paleness or change of skin color [9]. Because of the economic, social, and other negative consequences, anemia is a priority nutritional problem in most of the developing world [2,10-12].

The World Health Organization (WHO) estimates that the highest proportion of individuals affected by anemia are in Africa and that in Ethiopia anemia is a severe problem for both pregnant (62.7%: 95% CI 30.1-86.7) and non-pregnant women of childbearing age (52.3%: 95% CI 24.9-78.4) [2]. A few reports among the rural and urban communities in Ethiopia recognize anemia, particularly iron deficiency anemia, as a moderate public health problem in the country [13-17]. However, despite some available evidence [15,16,18-22], iron deficiency anemia was documented as a rarity in Ethiopia by some investigators who based their conclusion on small samples [23-25]. These investigators suggested that because of exposure to high iron intake [24,25] combined with hypoxia due to high altitude [24] and infestations with intestinal parasites or other illness [23] anemia was not due to malnutrition. The idea of the problem being rare persists among some researchers despite the age of the previous research and provides motivation for this study.

Given the fact that Ethiopia is among the poorest country in Africa with high rates of food insecurity and malnutrition [26,27] one may assume problems with iron deficiency anemia. Although Ethiopia has a wide range of agro-climatic conditions and grows a variety of cereals, root crops and vegetables, some of these are not fully utilized. There appears to be dependency on a single food crop by region although the specific crop varies in the different regions. The staple crops consumed in the North and Central part of Ethiopia are teff (Eragrostis tef) and cereals; in the South and Southwest staple crops are enset (Ensete ventricosum), cassava (Manihot esculenta), maize (Zea mays), cereals and root crops; and in the East staple crops are sorghum and maize. The lack of dietary diversity results in a shortage of minerals and vitamins which suggests that the bio-availability of much of the iron in the average Ethiopian diet is restricted and this restriction presumably affects the iron status [13]. The purpose of this study was to determine the prevalence of anemia, iron deficiency, and iron deficiency anemia in Ethiopian women of childbearing age using a large sample of the population.

## Methods

### Design

This paper is based on the first large nutrition and health study of a representative sample of women of reproductive age in nine of the eleven regions of Ethiopia: Tigray; Affar; Amhara; Oromiya; Benishangul-Gumuz; Southern Nations, Nationalities and Peoples (SNNP); Harari regions; Addis Ababa and Dire Dawa city administrations. Gambella and Somali regions were inaccessible at the time of the study for security reason and were not included. The study was conducted from June to July 2005 and included a total of 27,000 women of childbearing age drawn via a stratified cluster sampling method. The present study includes the systematically selected sub-sample of women who were assessed for biological parameters. The study was approved by the Research and Ethical Clearance Committee of the Ethiopian Health and Nutrition Research Institute (EHNRI). Prior to data collection, a thorough explanation was given to all subjects and informed verbal/written consent was obtained.

The health workers who collected data were drawn from the respective regions and trained to standardized methods using structured protocols and a pre-tested questionnaire. Nurses interviewed subjects and doctors performed the clinical evaluations. Biological samples were collected aseptically by lab technicians and analyzed partly at the site (stool and hemoglobin tests) and the results were documented in the respective questionnaire. All necessary safety measures were taken during blood collection. At the end of biological sample collections, all the subjects found positive for intestinal parasites were treated free on the spot at the expense of the project and incentives were given to the women while anemic cases were referred to the respective health institutions.

To maintain data quality, experienced supervisors from the Ethiopian Health and Nutrition Research Institute (EHNRI) checked the questionnaires on the spot to ensure completion and accuracy. Incorrect, unacceptable, and doubtful responses were assessed again the same day. Every evening survey group had meeting to discuss the experience of the day and plan for the next day. The completed questionnaires and data records were entered into a database at EHNRI. Details of the study methodology are presented in the technical report submitted to the donors [28].

### Subjects

To draw the full sample of 27,000 subjects, in each of 270 clustered villages (a cluster is equivalent to the smallest administrative unit as defined by the Government and is commonly known as "kebele"), one site was randomly selected and all women aged from 15 to 49 years were invited to participate in the study. Multistage cluster sampling methods were then applied to select the 100 women per cluster who were enrolled for the clinical assessment using probability proportional to the population size (PPS). To accomplish this, in each regional state, cumulative populations were calculated and attributed numbers assigned. The sampling interval was then calculated by dividing the total number of study population with the number of clusters. A random number was drawn using a random number table and the first cluster was selected based on this number. To select the remaining clusters, the sampling interval was added sequentially to the random number until all the 270 clusters, representing over 95 percent of the country, were selected. From these subjects (full sample) who were examined for clinical anemia or pallor, an approximately 5% sub-sample of 1135 women were systematically selected (starting from a random number, every nth subject) to be further assessed for socio-demographic, dietary, and biochemical variables. There was an inadequate amount of blood for analysis for 165 of the women, thus, 970 of the subjects with complete data for hemoglobin, serum ferritin, and diet were used in this analysis of iron deficiency anemia.

### Data collection

Dietary data were collected using a simplified food frequency questionnaire (FFQ) modified from the Helen Keller International FFQ that was used previously in Ethiopia, to estimate meat and vegetable consumption that was in addition to the staple food intake [29]. FFQ's that reliably estimate energy and precise nutrient intake are just being validated for Ethiopia but were not available at the time of this study [30]. Twenty common foods, irrespective of the staple food, were included on the FFQ (plant sources: banana, beans, bread, broccoli, cabbage, cassava leaves, ground nuts, morinaga, oranges, peanuts, potato, rice, spinach, Swiss chard; meat sources: beef meat, eggs, fish, liver, milk, poultry; and an "other" option). Responses were grouped according to frequency of meat and vegetables consumption at once per week or more for meat and once per day or more for vegetables. These are low thresholds for reasonable consumption from a nutritional perspective, but this pattern of intake was established based on the economic and social norms of the country.

All biological assays were done in duplicate and results were averaged. Hemoglobin concentration was measured at each study sites by trained medical technologists from capillary blood using a portable HemoCue photometer (HemoCue AB, Ängelholm, Sweden), which is considered to be a gold standard for field work [9]. Immediate feedback was given to the subjects including treatment of intestinal parasites with mebendazole when necessary. Anemia was defined as hemoglobin < 11 g/dL in pregnant women and < 12 g/dL for non-pregnant women [31,32] adjusted for altitude.

For the serum ferritin, venous blood was collected aseptically from the antecubital veins and alliquoted into tubes without anticoagulants. The serum was separated and stored frozen at -20°C and transported to the EHNRI for later determinations. Serum ferritin was measured by a fully automated Elecsys 1020 using commercial kits supplied by Boerrhinger Mannheim, Germany at EHNRI by a senior medical technologist. The high ferritin cut-off point (SF < 15 μg/L) recommended by WHO for developing countries was used to define iron deficiency in order to compensate for the effect of infection, which can lead to elevation of the level of ferritin [31,32]. Controls for the various concentrations ranges were run as a single determination at least once every 24 hours, once per repeated kit, and after every calibration.

### Statistical Analysis

Data were analyzed using Statistical package for the Social Science (SPSS) version 12. Standard tabulations were generated in which any outliers were identified prior to subjecting the data to analyses; ten cases were excluded as outliers. The chi-square test was used to test the association of anemia with various socio demographic and important health variables. Stepwise backward logistic regression model was also applied to test the association of intestinal parasites and type of illnesses in particularly to see their impact on iron absorption. P < 0.05 was considered statistically significant for all analyses.

## Results

### Subject characteristics

The mean (SD) age was 25 (9) years ranging from 15 to 49 years. The vast majority, 80%, were from rural settings. Slightly over a third of the women were engaged in mixed farming for an occupation while another third were civil servants or engaged in trade or handicrafts. Most of the women, 58.8%, had some formal education, 82.2% were married, 58.5% lived in a nuclear family, and 74.0% had birth spacing of more than 2 years. 9.7% of the subjects were pregnant (Table 1).

**Table 1:** Socio-demographic characters of the women in nine regions of Ethiopia, 2005

**Characteristic**		**n (%)**
Age, years		
	15-20	57 (5.9)
	21-30	138 (14.2)
	31-35	243 (25.1)
	36-40	173 (17.8)
	41-45	176 (18.1)
	46-49	183 (18.9)

Occupation		
	Mixed farming	351 (36.2)
	Livestock	33 (3.4)
	Trade	125 (12.9)
	Civil servant	154 (15.9)
	Factory worker	26 (2.7)
	Handicraft	40 (4.1)
	Others (i.e., housewife)	241 (24.8)

Settings		
	Rural	776 (80.0)
	Urban	194 (20.0)

Formal Education1		
	Yes	570 (58.8)
	No	400 (41.2)

Marital status		
	Married	797 (82.2)
	Divorced/widowed	173 (17.8)

Pregnant		
	Yes	94 (9.7)
	No	876 (90.3)

Family size		
	1-5	567 (58.5)
	>5	403 (41.5)

Birth spacing, years		
	≤ 2	252 (26.0)
	> 2	718 (74.0)

^1 ^Primary education and above; literate.

### Anemia

The proportion of women with anemia, iron deficiency, and iron deficiency anemia (IDA) is shown in Table 2. The mean (SD) hemoglobin was 11.5 g/dL (2.1) ranging from 9.0 to 13.5 g/dL with an overall prevalence rate of anemia of 29.4%. The World Health organization (WHO) states when percent of population with hemoglobin less than the above cut-offs points is 1-9%, 10-39% and greater than 40% within a population indicates mild, moderate and severe or high public health problem [7]. Accordingly, the prevalence is categorized as a moderate public health problem.

**Table 2:** Anemia, iron deficiency, and iron deficiency anemia prevalence by age in Ethiopian women

**Age**	**N**	**Anemia**^**1**^ **N (%)**	**Iron deficiency**^**2**^ **n (%)**	**Iron deficiency anemia**^**3**^ **n (%)**
15-20	57	11 (3.0)	10 (3.2)	6 (3.5)

21-30	138	23 (7.5)	25 (8.0)	13 (7.4)

31-35	243	75 (27.1)	87 (27.9)	51 (29.2)

36-40	173	54 (19.2)	60 (19.3)	31 (17.7)

41-45	176	55 (19.5)	59 (18.9)	37 (21.1)

46-49	183	67 (23.6)	70 (22.6)	37 (21.1)

Total	970	285 (29.4)*	311 (32.1)**	175 (18.0) ***

^1 ^Hemoglobin < 11 g/dL in pregnant women and < 12 g/dL for non-pregnant women.

^2 ^Serum ferritin < 15 ug/L.

^3 ^Hemoglobin < 12 g/dL and serum ferritin < 15 μg/L.

* = x^2 ^= 11.0; p = 0.04.

** = x^2 ^= 13.0; p = 0.02.

*** = x^2 ^= 8.89; p = 0.11 by age.

The mean (SD) serum ferritin level was 58 μg/L (41.1) ranging from 18 to 98 μg/L with an estimated prevalence rate of iron deficiency of 32.1%. The overall prevalence rate of iron deficiency anemia (hemoglobin <12 g/dL and serum ferritin <15 μg/L) was 18.0% (Table 2). The prevalence of all types of nutritional anemia among the age groups between 31-49 years was higher than the age group 15-20 and 21-30 years in descending orders. The differences noted by age were statistically significant for anemia, iron deficiency, and iron deficiency anemia nutrient deficiencies (p < 0.05).

The proportion of anemia was significantly higher among those women whose occupation was livestock rearing (p = 0.01). Those whose livelihood was primarily animal rearing might consume milk and its products that contain calcium which might have negatively impacted on their iron absorption (Table 3).

**Table 3:** Association of dietary and health variables with prevalence of anemia in Ethiopian women

**Variable**		**Total** **n (%)**	**Normal** **n (%)**	**Anemia**^**1**^ **n (%)**	**X**^**2**^	**P**
Occupation						
	Mixed farming	351 (36.2)	266 (75.8)	85 (24.2)	21.7	0.001
	Livestock	33 (3.4)	14 (42.4)	19 (57.6)		
	Trade	125 (12.9)	85 (68.0)	40 (32.0)		
	Civil servant	154 (15.9)	108 (70.1)	46 (29.9)		
	Factory worker	26 (2.7)	15 (57.7)	11 (42.3)		
	Handicraft	40 (4.1)	29 (72.5)	11 (27.5)		
	None(housewife)	241 (24.8)	158 (65.6)	83 (34.4)		

Type of family planning						
	None	521 (53.7)	353 (67.8)	168 (32.2)	15.8	0.003
	Pills	146 (15.1)	109 (74.7)	37 (25.3)		
	IUCD	7 (0.7)	5 (71.5)	2 (28.5)		
	Injection	285 (29.4)	203 (71.2)	82 (28.8)		
	Others (condom)	25 (2.6)	9 (36.0)	16 (64.0)		

Type of illnesses^**2**^						
	Pneumonia	39 (4.0)	25 (64.1)	14 (35.9)	16.7	0.001
	Malaria	42 (4.3)	26 (61.9)	16 (38.1)		
	Chronic illnesses^3^	45 (4.6)	20 (44.4)	25 (55.6)		
	None	844 (87.0)	604 (71.6)	240 (28.4)		

Intestinal parasites						
	Present	837 (86.3)	620 (74.1)	217 (25.9)	3.07	0.07
	Absent	163 (13.7)	123 (75.5)	40 (24.5)		

Vegetable consumption daily						
	Once a day or more	567 (58.5)	451 (79.5)	116 (24.5)	16.6	0.001
	Less than once a day	403 (41.5)	274 (68.0)	129 (32.0)		

Meat consumption weekly						
	Once a week or more	194 (20.0)	116 (59.8)	78 (40.2)	13.6	0.001
	Less than once a week	776 (80.0)	349 (45.0)	427 (55.0)		

^1 ^Hemoglobin < 11 g/dL in pregnant women and < 12 g/dL for non-pregnant women.

^2 ^Includes acute febrile illnesses and chronic infections such as tuberculosis.

^3 ^Chronic illnesses include TB, and persistent diarrhea.

### Diet

Over half, 58.5%, of the subjects reported consumption of vegetables at least once daily and 20% consumed meat at least once weekly (Table 3). For the subjects who ate vegetables and meat less frequently the occurrence rate of anemia was significantly higher (vegetables 32% vs. 24.4%, and meat 55.2% vs. 40.2%) than for their counterparts (p = 0.001) but the association was not retained in the hierarchical analysis (AOR = 1.1; 95% CI 0.74 - 1.1) (data not shown).

### Parasites and Illness

**Figure 1:**
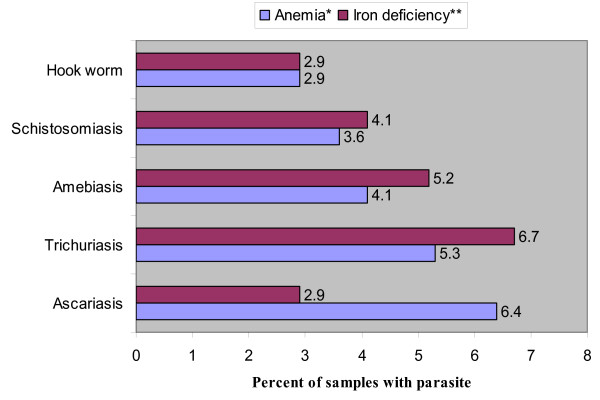
**Associations of intestinal parasites with occurrences of anemia and iron deficiency in Ethiopian women**. * = x^2 ^= 0.01; p = 0.9. ** = x^2 ^= 0.01; p= 0.9

Of the 970 subject, 837 of them had either single or multiple parasites which led to the increase in the overall number of parasites to 1187. The proportion of Ascariis lumbricoids, Trichuris trichiura, Entameba histolithica, Schistosoma mansoi and Hook worm or Ancylostoma duodenale was 23.6% (280/1187), 19.0% (226/1187), 15.1% (179/1187), 12.9% (154/1187) and 11.0% (131/1187) respectively indicating that the major types of intestinal parasites were Ascariasis and Trichuriais. The association of intestinal parasites was further investigated with the occurrence of anemia after the presence of parasites was dichotomized. Of the 837 subjects who had parasites, only 25.9% (217/837) had anemia and the differences noted was not statistically significant (P = 0.07) (Table 3). When the association of anemia was further disaggregated by parasite types, the most common parasite observed was Ascariasis, 7.4% (62/837), followed by Trichuriasis 6.1% (51/837), Amebiasis 4.8% (40/837), Schistosomiais 4.2% (35/837) and Hook worm infestations 3.5% (29/837) suggesting that intestinal parasite in the present study was less likely to be the causative agent of anemia (Figure 1).

The prevalence of anemia was associated with history of illnesses, and the differences noted were significant (p = 0.001). When the association of anemia was further analyzed (in the logistic regression models) by type of illnesses, there were significant differences in the type of illness distributed as follows: pneumonia (p = 0.3), malaria (p = 0.1), and chronic illnesses (p = 0.001) (AOR = 0.3; 95%CI = 0.17 to 0.58) (data not shown) signifying that the most probable causes of anemia is nutrition related and to some extent chronic illnesses.

## Discussion

The criteria for determining the presence of anemia, as recommended by the World Health Organization (WHO), are based on hemoglobin cut-off values for age and sex with an additional epidemiological criterion for assessing the severity and magnitude of the problem in populations [1,2,6,7]. Ideally, prevalence studies should be based on a representative sample composed of every segment of population but when this is not feasible, the prevalence in high-risk group could be a valid indication for the magnitude of the problem [5]. Based on such premises, in Ethiopia a few studies with small sample size conducted at different times documented the existence [9,13-22,33] of iron deficiency anemia while other studies reported it as being rare in the country [23-25] despite the fact that iron deficiency anemia remains a widespread public health problem in most developing countries and even developed countries, a considerable number of women tend to have inadequate reserve to meet the requirement of pregnancy. [34]

Estimates of iron deficiency anemia (IDA) in our study, 18.0%, were based on two measures, hemoglobin and serum ferritin, and indicate a moderate public health problem according to WHO standards [7]. This is similar to a prevalence of IDA of 22.3% in a group of 197 lactating women in Addis Ababa, Ethiopia [18] and 18.4% in a group of 1134 pregnant and lactating women in Ethiopia [35] based on these same measures. Additionally prevalence of anemia was 15.1% based on low hemoglobin in a cross-sectional survey of 403 pregnant women attending an urban health centre in Awassa, (Southern Ethiopia) [9], and 13% of a group of 99 women in late pregnancy from Sidama (Southern Ethiopia) based on mean cell hemoglobin concentrations and mean corpuscular volumes [17]. The concordance of these relatively recent estimates points to the need to recognize and address iron deficiency anemia in women of childbearing ages in Ethiopia. While it is possible that some of the approximately 11% of women with anemia that does not appear to be from iron deficiency might have elevated ferritin levels as an acute phase protein or hemoglobinopathy, it is more likely that other micronutrient deficiencies are the problem. Vitamin A is a major public health problem in both preschoolers (prevalence of 1.5 Bitot's spots) and mother (night blindness of 2 - 15%) in Ethiopia. Furthermore, folic acid is another nutrient deficiency observed among women of reproductive age [6,11,26,33].

Although some crops, notably teff, are high in iron [26] and fermented enset may increase non-heme iron absorption [36], a marked observation, in slightly more than half of the subjects with anemia, in the present study is the low intake of meat which is a source of heme iron. Heme iron is not only better absorbed than non-heme obtained from plant source food, whose absorption may range from 1-10%, but also has an enhancing effect on absorption. The finding that about one-third of the women with anemia in this study had vegetables less than once a day suggests low consumption of vitamin C and could be another substantiating factor for the existence of iron deficiency anemia. This dietary pattern would also result in low levels of zinc which was found to be a strong predictor for hemoglobin in pregnant women in Southern Ethiopia [17]. A study with children in Ethiopia [15] which included a thorough assessment of dietary intake showed that dietary iron was adequate but bioavailability was restricted because the type of iron was non-heme and there was inadequate vitamin C, additionally, absorption was further reduced due to the presence of inhibitory factors such as tannins, phenols, and fiber [15]. These findings and ours strongly suggest that iron deficiency anemia in Ethiopia can be explained by dietary factors.

Despite the various types of nutrition interventions that have been implemented in Ethiopia, cultural food taboos and religious fasts persist among different ethnic, religious, age and gender groups. Taboos have limited the development and utilization of certain plant and animal food resources, with detrimental effect particularly on mothers and children. Camel meat and milk are avoided by Christians and many pagan people in the south traditionally avoid chicken and eggs while the Oromo, Afar, Somali, and other pastoralist groups avoid fish. The rejection of fish and most wild animals as food has persisted in most pastoralist groups. Aversion to eating ensete (Ensete ventricosum) and the meat of pigs and goats spread with the southward migrations of the northern Ethiopians. In addition, food avoidance in the form of reluctance to eat vegetables and fruit are widespread in grain growing areas [33]. Increasing animal food consumption through poultry rearing and small ruminants [13] and intensification of backyard gardening to ensure availability and increased consumption of vegetables and fruits is recommendation to partly address the problem.

As expected, due to cumulative obstetric conditions and maternal exhaustions, the prevalence of anemia among the age groups between 31-49 years was higher than the age group 15-20 and 21-30 years in descending orders. Such observations are not uncommon to see in most sub-Saharan and African countries [5,17]. Women frequently enter pregnancy with insufficient nutrient stores, and thus the increased demand associated with pregnancy and later with lactation is reported to cause anemia [7,34]. This holds true in most African countries and Ethiopia could not be the exception given the high rates of micro and macronutrient deficiencies resulting from the interaction of deficient dietary intake and the deeply entrenched food habits [13,33].

Although the presence of parasitic infections, particularly hookworm, is associated with bleeding and higher levels of iron deficiency [2] this was not found in our study which again substantiates our assertion that iron deficiency anemia is mainly caused by dietary factors in Ethiopia. Nonetheless, anemia was significantly higher in women with history of illness and the association was retained even when the variable was adjusted for its confounding effect in the logistic regression models signifying that another cause of anemia to some extent is chronic illnesses as well.

A limitation to this study is lack of more precise estimates of nutrient intake due to the methods of dietary assessment. Further any data based on recall such as the food frequency information is subject to bias as well. Also, it is possible that a bias was introduced due to missing data since not all women selected systematically for the biological collections had complete blood data. Limitations such as these are common challenges when doing field work with large samples.

## Conclusion

This study is the first of a large sample of women from nine of the eleven regions in Ethiopia and reveals an overall prevalence of iron deficiency anemia that can be considered a health problem. The dependency on a single staple crop results in shortage of minerals and vitamins which may lead to iron deficiency anemia and therefore should be addressed within the country. Additionally, weekly iron-folic acid supplementation in areas where the prevalence of anemia exceeds 20% to vulnerable groups particularly the pregnant and lactating women together with health education is warranted to achieve several of the United Nation's Millennium Development Goals. Although anemia can be caused by non-nutritional factors [3,4] in our study there was no association between parasitic infestations and anemia. However, chronic illnesses such as TB and probably HIV were found statistically significant in both bi-variate as well as the logistic regression models [13,17] suggesting that chronic illness could be the second important causes for anemia in women of childbearing ages in Ethiopia.

## Competing interests

JAH was formerly employed with the Ethiopian Health and Nutrition Research Institute of the Federal Ministry of Health. Otherwise, the authors declare that they have no competing interests.

## Authors' contributions

JAH organized this study when he was working for the Ethiopian Health and Nutrition Research Institute, conducted statistical analysis, and drafted the manuscript. RSP contributed to data interpretation and revising the manuscript for intellectual content. Both authors read and approved the submitted manuscript.

## Pre-publication history

The pre-publication history for this paper can be accessed here:


